# Stimulation of Single, Possible CHX10 Hindbrain Neurons Turns Swimming On and Off in Young *Xenopus* Tadpoles

**DOI:** 10.3389/fncel.2019.00047

**Published:** 2019-02-18

**Authors:** Wen-Chang Li, Stephen R. Soffe

**Affiliations:** ^1^School of Psychology and Neuroscience, University of St Andrews, St Andrews, United Kingdom; ^2^School of Biological Sciences, Tyndall Avenue, University of Bristol, Bristol, United Kingdom

**Keywords:** central pattern generator, swimming, excitatory interneurons, spinal cord, hindbrain, CHX10

## Abstract

Vertebrate central pattern generators (CPGs) controlling locomotion contain neurons which provide the excitation that drives and maintains network rhythms. In a simple vertebrate, the developing *Xenopus* tadpole, we study the role of excitatory descending neurons with ipsilateral projecting axons (descending interneurons, dINs) in the control of swimming rhythms. In tadpoles with both intact central nervous system (CNS) and transections in the hindbrain, exciting some individual dINs in the caudal hindbrain region could start swimming repeatedly. Analyses indicated the recruitment of additional dINs immediately after such evoked dIN spiking and prior to swimming. Excitation of dINs can therefore be sufficient for the initiation of swimming. These “powerful” dINs all possessed both ascending and descending axons. However, their axon projection lengths were not different from those of other excitatory dINs at similar locations. The dorsoventral position of dINs, as a population, significantly better matched that of cells marked by immunocytochemistry for the transcription factor CHX10 than other known neuron types in the ventral hindbrain and spinal cord. The comparison suggests that the excitatory interneurons including dINs are CHX10-positive, in agreement with CHX10 as a marker for excitatory neurons with ipsilateral projections in the spinal cord and brainstem of other vertebrates. Overall, our results further demonstrate the key importance of dINs in driving tadpole swimming rhythms.

## Introduction

Vertebrate locomotion is directly controlled by the neuronal circuits in the spinal cord and brainstem. The conventional concept is that the spinal cord contains the basic circuit, termed central pattern generator (CPG), that can generate the locomotor rhythms by transforming excitatory commands descending from supraspinal centers like the reticulospinal nuclei (Dubuc et al., 2008; Kiehn, 2016). More recent studies in tadpoles (Li et al., 2006; Soffe et al., 2009), larval zebrafish (Kimura et al., 2013) and lamprey (Buchanan, 2018) suggest that the swimming CPG network may extend into the brainstem. Among CPG neurons, glutamatergic excitatory neurons, especially those with ipsilateral projecting axons, have been shown to play cardinal roles in locomotion rhythm generation of both limbed and non-limbed animals (Jordan et al., 2008; Goulding, 2009; Roberts et al., 2010; Kiehn, 2016). In *Xenopus* tadpoles, as in lamprey, excitatory interneurons with ipsilateral projecting axons have been long identified in the CPG for axial swimming by their anatomy and physiology (Dale and Roberts, 1985; Dale and Grillner, 1986). Tadpole descending interneurons (dINs) in the caudal hindbrain and rostral spinal cord have been shown to be excitatory by coreleasing both glutamate and ACh (Li et al., 2004b). They form feedback excitatory connections among themselves (Li et al., 2006), are electrically coupled (Li et al., 2009) and their firing leads the activity of all other swimming CPG neurons (Soffe et al., 2009). Injecting large currents into dINs can change swimming frequencies (Li and Moult, 2012) and silencing dINs using large hyperpolarizing current injections can stop swimming within milliseconds (Moult et al., 2013). Although they are most easily recorded in the caudal hindbrain and rostral spinal cord region and there are reliable physiological and anatomical criteria to identify them, a molecular marker for the dIN population is still lacking.

The expression pattern of various transcription factors during early development has been used to trace the origin and to classify several groups of excitatory neurons (Goulding, 2009; Gosgnach, 2011; Kiehn, 2016). In mice, this has allowed manipulation of the function of these genetically identified groups of neurons to interrogate their roles in locomotor control. The V2a group of excitatory interneurons express ceh-10 homeodomain containing homolog (CHX10; Lundfald et al., 2007; Crone et al., 2008). Ablating V2a neurons affected the left-right alternation of locomotor rhythms in the high frequency range (Crone et al., 2008, 2009) and their role does not seem to be rhythm-generating (Kiehn, 2016). Ablation of V2a interneurons in the cervical spinal cord only disrupts forelimb reaching (Pivetta et al., 2014; Hayashi et al., 2018). The expression of short stature homeobox protein 2 (SHOX2) defines another excitatory interneuron group with ipsilateral axons (Dougherty et al., 2013), which partially overlaps with V2a CHX10-expressing neurons. V2a SHOX2+ neurons directly excite motoneurons (MNs) and the nonV2a SHOX2+ neurons may be part of the mammalian locomotor CPG. Neurons expressing basic helix-loop-helix domain containing, class B, 9 (HB9) transcription factor may also be part of the excitatory drive in mammalian CPG (Hinckley et al., 2005; Wilson et al., 2005), controlling the frequency of locomotion (Caldeira et al., 2017). These studies clearly show excitatory neuron types in mice belong to a number of diverse functional groups.

Transcription factor expression has also been studied in developing zebrafish where CHX10-expressing glutamatergic V2a interneurons (CiD) extend from the spinal cord into the hindbrain (Kimura et al., 2006, 2013). Optically exciting V2a neurons in the hindbrain could initiate swimming and inhibiting them could stop ongoing swimming in zebrafish larvae (Kimura et al., 2013). In the spinalized preparation, exciting V2a neurons could also induce fictive swimming bouts (Ljunggren et al., 2014). V2a firing during swimming in the hindbrain is phasic in the middle hindbrain region and more tonic in the caudal hindbrain, thus likely providing both tonic and phasic excitation in the swimming network (Eklöf-Ljunggren et al., 2012; Kimura et al., 2013). The anatomical and functional evidence strongly suggests that dINs in the tadpole swimming circuit are similar to V2a neurons identified in larval zebrafish (Kimura et al., 2006, 2013; Ljunggren et al., 2014) and might also, therefore, be expected to be of CHX10 origin.

In this study, we show that the activation of some individual, powerful dINs was sufficient to start swimming. We analyze the anatomical features of these neurons and show that their distribution suggests that they are, indeed, likely to be of CHX10 origin.

## Materials and Methods

*Xenopus laevis* tadpoles were raised from fertilized eggs after inducing mating between pairs of adult male and female by injecting human chorionic gonadotropin solution into the dorsal lymph sac. All experimental procedures were approved by the local Animal Welfare and Ethics Committee at the University of St Andrews and the University of Bristol and comply with UK Home Office regulations. At stage 37/38, ~2 days post fertilization, tadpoles were briefly anesthetized with 0.1% MS-222 (3-aminobenzoic acid ester, sigma, UK). Then the tadpole was pinned down onto a Sylgard-lined stage in a bath to have its dorsal fin cut open with a fine tungsten needle. Saline contained (in mM): NaCl 115, KCl 3, CaCl_2_ 4, NaHCO_3_ 2.4, MgCl_2_ 1, HEPES 10, with pH adjusted to 7.4 using 5 M NaOH. Next the animal was left in 10 μM α-bungarotoxin saline for immobilization. Afterwards, it was re-pinned onto the Sylgard stage to allow further dissections, which included removing some dorsal trunk skin and muscles over the spinal cord, exposing rostral spinal cord and caudal hindbrain, cutting open the dorsal roof of the exposed central nervous system (CNS) and removing some ependymal cells inside the spinal cord and hindbrain to provide access to neuronal somata for recordings.

Single or dual whole-cell patch clamp recordings were made in current clamp mode. Patch-clamp pipettes were filled with 0.1% neurobiotin in an intracellular solution (K-gluconate 100 mM, MgSO_4_ 2 mM, EGTA 10 mM, HEPES 10 mM, Na_2_ATP 3 mM, NaGTP 0.5 mM) and had resistances of ~10 MΩ. Neurobiotin filling of neurons was achieved by passive diffusion during recordings. After experiments, tadpoles were fixed in 2% glutaraldehyde in 0.1 M phosphate buffer (pH 7.2) overnight in the fridge. After rinsing with 0.1 M phosphate buffered saline (PBS), tadpoles were treated with two changes of 1% triton-X100 in PBS for 15 min with agitation. Then they were incubated for 2–3 h in a 1:200 dilution of extravidin peroxidase conjugate (Sigma) in PBS containing 0.5% Triton-X100 with agitation. The animals were washed afterwards with a few changes of PBS. Then they were pre-soaked in 0.08% diaminobenzidine in PB (DAB solution) for 5 min and moved to a second pot with 0.03% hydrogen peroxide in DAB solution for five more minutes. The staining reaction was stopped by washing the preparations with tap water. Further dissections were carried out to free the brain and spinal cord with the notocord and some ventral muscles for slide-mounting using Depex after dehydration with alcohol and clearance with methyl benzoate and xylene.

For CHX10 staining, tadpoles were fixed in MEMFA (3.7% formaldehyde, 0.1 M MOPS, 2 mM EGTA, 1 mM MgSO_4_) for 2 h, rinsed in two changes of methanol and stored overnight in methanol at −20°C. The spinal cord was exposed by dissection following rehydration and the tissues bleached in 15% hydrogen peroxide in 0.1 M PBS for 2 h. Then specimens were washed with three changes of PBT (PBS with 0.1% Triton X100 and 20 mg/ml bovine serum albumin) for 15 min each and then blocked in 10% normal goat serum in PBT. They were next transferred to primary antiserum CHX10 (1:1,000, kindly provided by Dr. Martyn Goulding) for 72 h, washed 5× 1 h in PBT, incubated overnight in secondary antibody (peroxidase conjugated F(ab)2 fragment goat anti-rabbit IgG (Jackson Immunoresearch, with 1:500 dilution in PBT). After washing 5× 45 min in PBT, the peroxidase was visualized using nickel-enhanced diaminobenzidine with glucose oxidase to generate the hydrogen peroxide. After two washes in PBS, specimens were cleared in Murrays Clear (2:1 Benzyl benzoate:benzyl alcohol) and mounted between coverslips.

Neurons were observed using a ×100 oil immersion objective to check the axon trajectories. Axons and soma positions were traced using a ×20 objective. All measurements were corrected for shrinkage during dehydration by multiplying by 1.28. Data distribution was routinely checked before comparisons were carried out using IBM SPSS Statistics 24. For normally distributed data, *t*-test or one way ANOVA were adopted and for other datasets, non-parametric tests were used. Figures were prepared using CorelDRAW Graphics Suite X6.

## Results

**Figure 1:**
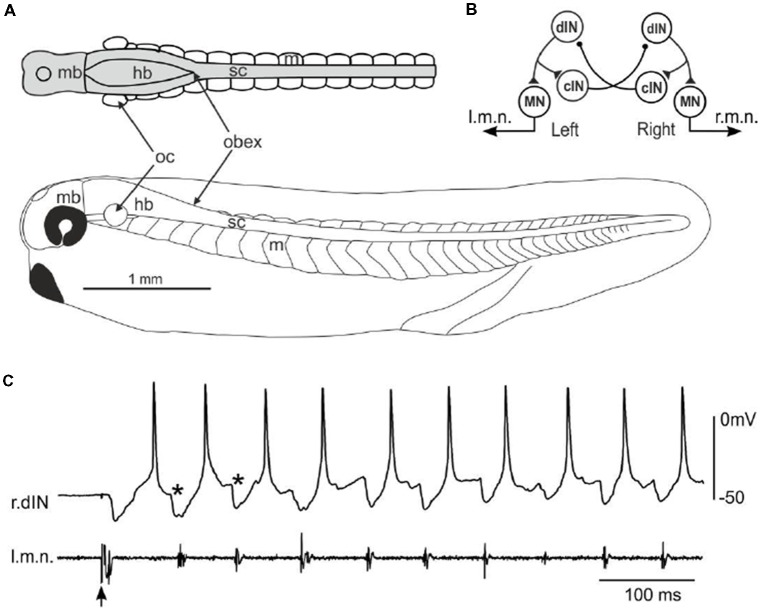
Fictive swimming in a stage 37/38 tadpole and the neural circuit controlling swimming. **(A)** A side-view scale diagram of a developing tadpole at stage 37/38 with a top view of its central nervous system (CNS; gray) showing: m swimming myotomes; mb, midbrain; hb, hindbrain; sc, spinal cord; oc, otic capsule. Obex is where the hindbrain ventricle closes and the landmark separating hb and sc. **(B)** Diagram showing most critical neuron populations and their synaptic connections in tadpole swimming central pattern generator (CPG). Each circle represents a neuron population. dIN, descending interneuron; cIN, commissural interneuron; MN, motoneuron. l.m.n. and r.m.n.: left and right motor nerve. **(C)** Typical activity of a right side dIN (r.dIN) and simultaneous left motor nerve (l.m.n) activity at the beginning of an episode of fictive swimming started by electrical stimulation to the head skin (arrow). *Indicates cIN inhibition.

Hatchling *Xenopus* tadpoles at stage 37/38 (Figure 1A) swim forward when they are briefly touched on the trunk skin. The neuronal circuit responsible for generating the swimming rhythms has been located in the caudal hindbrain and spinal cord, comprising four types of neurons including MNs, dINs, commissural interneurons (cINs) and ascending interneurons (aINs, Figure 1B, aINs not shown for simplicity). Neuronal activity can be recorded once the animals are immobilized using α-bungarotoxin and dissections made to expose neuronal somata. The fictive swimming frequencies (10–25 Hz) are very similar to those for free swimming (Roberts et al., 2000). During fictive swimming, CPG neurons like dINs typically fire a single action potential on each swimming cycle, with firing alternating between the left and right sides coordinated by reciprocal inhibitory coupling mediated by the cINs (Figure 1C). dINs can be physiologically identified by their single firing at the onset of step current injections, rebound-firing following phasic inhibition provided the membrane potential is held depolarized, their broad action potentials and one-spike-per-cycle firing in nearly all swimming cycles. Anatomically, dINs typically have ipsilateral descending axons and half of them in the caudal hindbrain and rostral spinal cord region also possess ascending branches (Li et al., 2006).

### The Initiation of Swimming by Single dINs in Tadpoles With Hindbrain Transections

The importance of populations of neurons in a neuronal network can be demonstrated by their sufficiency and necessity for network activity. We previously showed that blocking dIN activity by injecting large hyperpolarizing current into individual dINs could stop ongoing swimming (Moult et al., 2013) possibly by currents spreading to other dINs *via* their electrical coupling, suggesting dINs are essential in swimming rhythmogenesis. We also reported that spiking in one dIN evoked by current injection could often initiate swimming, but only if magnesium was omitted from the saline to increase excitability by turning off the voltage dependency of NMDAR-mediated excitation (Li et al., 2006).

**Figure 2:**
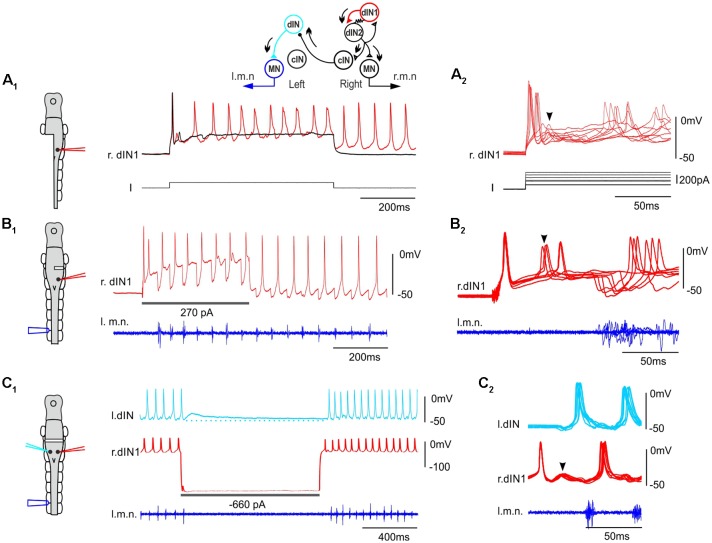
Stimulating single dINs in tadpoles with hindbrain transections starts fictive swimming. **(A_1_)** Step current injection into a right side dIN (r.dIN1) initiates swimming-like rhythms in a tadpole with the left side spinal cord and caudal hindbrain removed (black trace shows a failed trial). **(A_2_)** Nine superimposed trials as in **(A_1_)** but on a faster time scale and with different injected current levels. **(B_1_)** Injecting depolarizing currents into a dIN in a tadpole with its right side hindbrain transected starts swimming. **(B_2_)** Seven successful trials are overlapped on a faster time scale. **(C_1_)** The rebound spiking of a right side dIN (r.dIN1) following the withdrawal of a hyperpolarizing current injection, which has stopped swimming for 1 s, re-starts swimming in a dual whole-cell recording. Dotted line indicates the resting membrane potential of l.dIN. **(C_2_)** Eight superimposed trials. Traces are lined up to the rising phase of the first dIN spike in **(B_2_,C_2_)**. Diagrams on the left show CNS as in Figure 1A with location of lesions and electrodes (same color-coded as the recording traces). Arrowheads indicate secondary EPSPs and spiking following the initial dIN spiking. Inset shows sequence of activity in the CPG (arrows) with resistor sign representing electrical coupling among ipsilateral dINs.

We have now made recordings from over 200 dINs and confirmed our previous finding that stimulating a single dIN in intact tadpoles rarely initiates swimming in normal saline (containing 1 mM magnesium). However, under some circumstances stimulating single dINs to fire was able to elicit episodes of swimming. All were cases in which the spinal cord and hindbrain were (partially) disconnected from higher brain areas. When one side of hindbrain and spinal cord were removed, single spiking in 2/48 dINs evoked rhythmic activity in 13/44 trials (Figure 2A). In preparations where one side of hindbrain was transected at the otic capsule level, spiking in 1/39 dINs on the transected side initiated swimming multiple times (7/19 trials, Figure 2B). In addition, when the hindbrain was completely transected at the otic capsule level and large intracellular hyperpolarizing current injections were used to stop skin stimulation-evoked swimming in whole-cell dIN recordings (Moult et al., 2013), swimming could start again at the end of current pulse (1 s duration, swimming stopped for 400–1,000 ms) following rebound firing of the dIN (6/41 dINs, 45/97 trials, Figure 2C, depolarizing currents not tested). dINs typically receive tapering depolarization lasting for several seconds after swimming stops (for comparison, see hyperpolarization in other types of CPG neurons, Zhang and Sillar, 2012). A brief cessation of swimming would mean most dINs were still depolarized when the rebound spiking took place in the injected dIN, which should lower the swimming threshold. We measured the average depolarization levels in four non-injected dINs at the point of rebound spiking in paired recordings (Figure 2C_1_). They were 2.5, 3, 6.2 and 9.8 mV, despite being electrically coupled to dINs injected with large hyperpolarizing currents.

**Figure 3:**
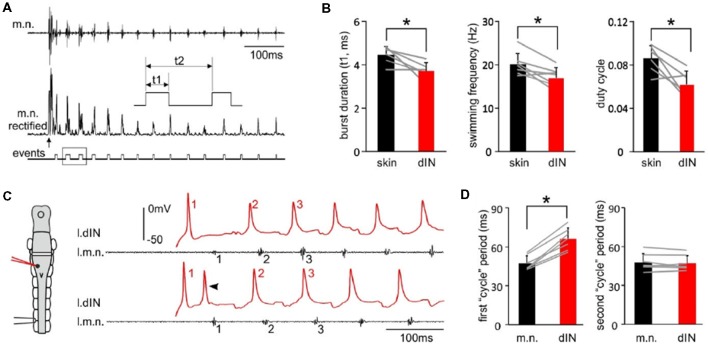
The features of swimming evoked by powerful dINs. **(A)** The beginning of a swimming episode started by tail skin stimulation (arrow). m.n. trace is rectified and threshold set to trigger burst events. Box area is stretched in the inset to show burst duration (t1), swimming frequency (1/t2) and duty cycle (t1/t2). **(B)** Swimming burst durations, frequencies and duty cycles for the first 50 cycles are lower in the episodes evoked by powerful dINs (red) than those started by skin stimulation (black). **(C)** The beginning of two swimming episodes started by a powerful dIN (traces before the initial spiking is off scale). Numbers in red and black mark dIN spikes and m.n. bursts used for calculating spiking/cycle periods, respectively. Both dIN and m.n. are recorded from the left side (diagram, l.dIN, l.m.n.). Arrowhead points at secondary dIN spiking. **(D)** The first, but not the second dIN spiking period is longer than swimming cycle period measured using m.n. bursts. Gray lines link measurements from the same recordings in **(B,D)**. *Indicates *p* < 0.05.

Tadpole swimming is normally initiated by the activation of the mechanosensory pathway innervating the skin. We first asked whether swimming started directly by these powerful, individual dINs differed from swimming initiated by sensory stimulation. We compared three swimming parameters, motor nerve (m.n.) burst duration, swimming frequency and duty cycle, in the first 50 cycles of swimming evoked by powerful dINs or by skin stimulation (Figure 3A). All three parameters were lower in swimming episodes started by seven powerful dINs, in that swimming frequency was lower and with relatively shorter motor bursts (*p* < 0.05, all paired *t*-test, Figure 3B). However, basic form of the alternating swimming pattern was the same. These data show that activation of dINs is sufficient in inducing swimming activity.

We have previously shown that dINs fire relatively early on each swimming cycle and their activity drives all CPG neuronal activity on the same side (Soffe et al., 2009). However, the delay between an evoked spike in a powerful dIN and the first swimming motor burst was clearly longer than the delay for subsequent cycles (compare numbered events in Figure 3C). To quantify this difference, we simply compared the periods of the first two cycles, measured separately for dINs and motor bursts (Figure 3D). As expected, these matched closely for the second cycle (dINs 47.3 ± 5.8 ms, motor bursts 48 ± 6.6 ms; *p* = 0.47, paired *t*-test, *n* = 7). However, for the first cycle, dIN period was significantly longer than motor burst period (dIN 66.1 ± 8.4, motor bursts 47.3 ± 5.6 ms; Wilcoxon matched-pairs signed-ranks test, *p* < 0.05, *n* = 7). The first motor burst was therefore 18.9 ± 5.4 ms later than expected from the delay between dIN spikes and motor bursts on subsequent cycles. This additional delay indicates that the initial dIN spiking does not directly drive the first motor burst, but that some further step is involved.

Then what happens after the initial dIN spiking to initiate swimming only after a relatively long delay? In each of the 65 trials where dIN spiking evoked swimming, some clear EPSPs followed the initial dIN spiking with a latency of 15 ± 2.7 ms (Figures 2A_2_,C_2_). These EPSPs gave rise to secondary spikes in the stimulated dIN in 19 trials (Figures 2B_2_, 3C). We previously revealed feedback excitation among dINs in the caudal hindbrain region (Li et al., 2006) and there was also widespread electrical coupling among dINs (Li et al., 2009). We suggest the most likely explanation for the events following an evoked dIN spike is that both types of synaptic connections act to recruit more dINs to amplify the initial excitation from this single spike. The sequence of event would therefore be that: the initial, evoked dIN firing would lead to excitation and recruitment of firing in a wider population of dINs; this in turn would produce the further EPSPs and secondary spiking seen in the recorded dIN; and firing across this wider dIN population would initiate the swimming rhythm (Figure 2, top diagram).

### The Initiation of Swimming by a dIN in a Tadpole With Intact CNS

**Figure 4:**
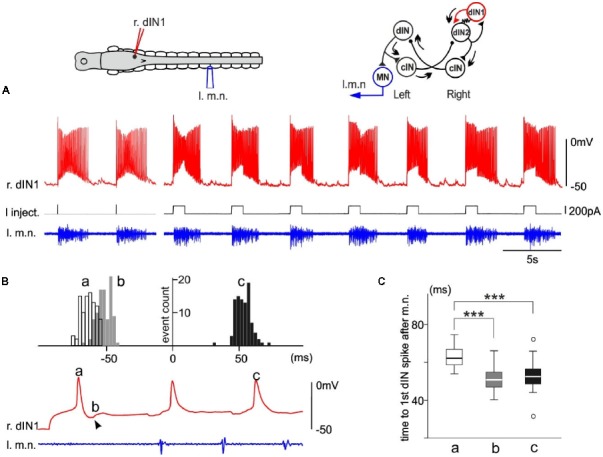
Single spiking in a right side dIN (r.dIN1) in a tadpole with intact CNS reliably evokes fictive swimming (right inset drawing with arrows showing activity sequence). **(A)** In nine trials (two 10 ms and seven 1,000 ms current pulses), r.dIN1 spiking initiates swimming. **(B)** The distribution of r.dIN1 spike and EPSP time relative to the first spike after the first l.m.n. bursts (time 0, 94 trials for the histogram). The timing of three events (see the trace below for examples) are measured: initial r.dIN1 spike evoked by current injections (“a,” unfilled histogram); onset of the first EPSP (“b,” arrowhead, gray); 2nd r.dIN1 spike after m.n. bursts (“c,” filled). **(C)** Comparison between the time of the three events in **(B)**. ***Indicates *p* < 0.001.

In one dIN from a tadpole with an intact CNS, swimming episodes were also reliably evoked by dIN current injections. From this example, we were able to estimate when the recruited dINs fired spikes by analyzing the timing of their EPSPs produced in the stimulated dIN. Injecting step currents of either 10 ms or 1,000 ms in duration evoked a single spike in this dIN and started swimming reliably (10/11 trials of 10 ms and 84/94 trials of 1,000 ms pulses, Figure 4A). Since the first two swimming cycle periods measured for motor bursts are similar (see above), we should expect similar spiking periods in the recruited dINs (dIN2, top right inset in Figure 4), which drive the motoneuron firing and motor nerve discharges. We measured the delay of three events relative to the first dIN spike after the first motor nerve burst (0 ms, Figure 4B): (a) the initial dIN spike; (b) onset of the first EPSP; and (c) the second dIN spike after the 1st motor nerve burst. In agreement with the analyses in Figure 3, event “a” had an average delay of 62.4 ± 5.1 ms, longer than the average delay of 52.7 ± 5.8 ms for event “c” (*p* < 0.001, *n* = 94, non-parametric median tests). The spike time for recruited dINs should be given by the delay for event “b” (50.1 ± 5.6 ms) plus the synaptic delay (~1 ms). The deduced spiking time of (51.1 ± 5.6 ms) for the recruited dINs was similar to the delay for event “c” (*p* = 0.13, *n* = 94, non-parametric median tests, Figure 4C) as we expected. Therefore, these analyses illustrate how the powerful dINs may have started swimming (top right inset in Figure 4), i.e., by recruiting more dINs on the same side to amplify the excitation.

### The Distributions and Axon Projections of Powerful dINs

**Figure 5:**
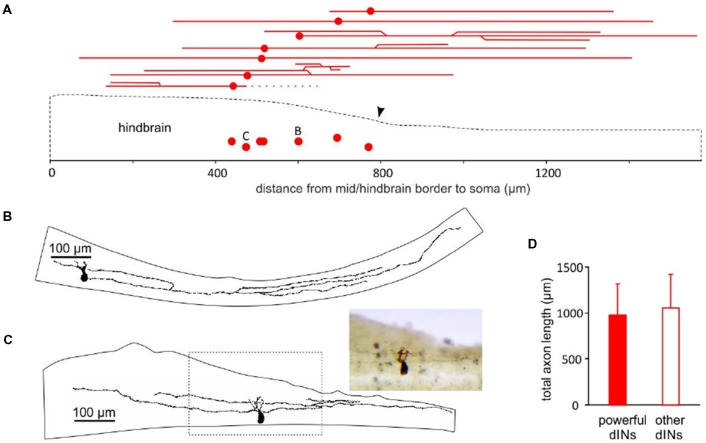
The location of powerful dIN somata and longitudinal axonal projections. **(A)** A diagram of the tadpole hindbrain and rostral spinal cord outline (dashed) showing the location of seven somata of the 10 powerful dINs in Figures 2, 3, 4 (red dots) relative to the mid/hindbrain border (0). The red lines above the sketch show the trajectories of their ascending and descending axons (dotted line indicates a broken axon). Arrowhead points at the obex. **(B)** Drawing of the axon branches and their trajectories in the CNS for dIN B marked in (**A**; also recording in Figure 2B). **(C)** Drawing of the axons and their trajectories for dIN C in (**A**; also recording in Figure 4). The area within the dashed box is photographed (inset). **(D)** Axon lengths of the seven powerful dINs are similar to those of other 12 dINs within the same region.

We previously reported that about half of the dINs in the caudal hindbrain and rostral spinal cord area had ascending axon branches in addition to their primary descending axon (Li et al., 2006). All of the nine powerful dINs recorded in tadpoles with hindbrain transections (Figure 2) and the 1 dIN recorded in an intact CNS (Figure 4) were located in the caudal hindbrain region. We traced the axon trajectories of seven of these dINs whose anatomy was revealed clearly by neurobiotin staining (Figure 5). All of the seven powerful dINs possessed both ascending and descending axons. Four of them had additional branches and more extensive axon branching patterns. These axons tended to project throughout the caudal hindbrain and rostral spinal cord. This was compared to the anatomy of 12 other dINs, the excitation of which had failed to initiate swimming, at similar locations in tadpoles with transections in the hindbrain (Figure 2C_1_, inset). Three of the 12 dINs did not have ascending axons and five of them possessed more than two axonal branches (3–6). Their total axon lengths (1,056 ± 367 μm) were similar to those of the seven powerful dINs (980 ± 340 μm, two-tailed independent *t*-test, *p* = 0.65, Figure 5D). The lack of distinctive anatomy of these powerful dINs suggests that they may have powerful synaptic excitation that allows them to evoke spiking in the remaining dINs in the swimming circuit to initiate swimming.

### Is CHX10 Expressed by dINs?

**Figure 6:**
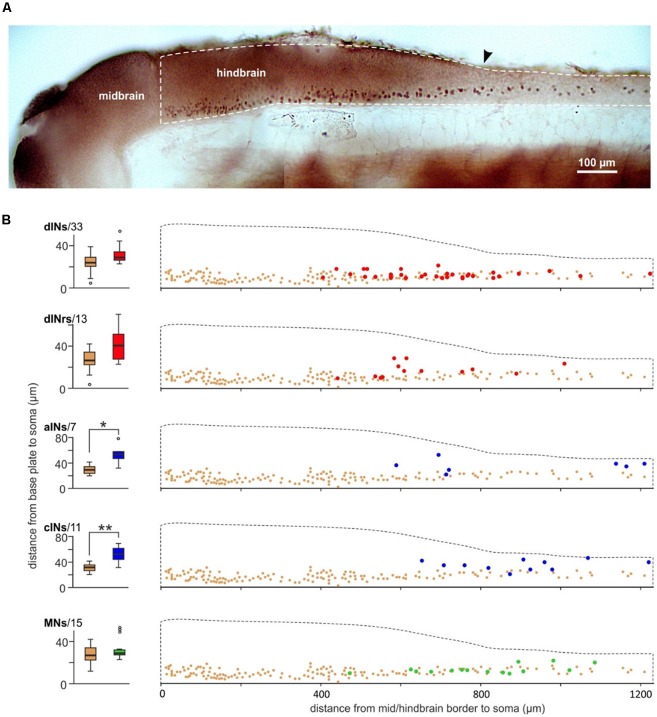
Comparing the location of CHX10 immuno-positive cells and neurons identified by their anatomy and physiology. **(A)** Photograph of CHX10 immuno-positive cells in the right side CNS of a stage 37/38 tadpole (image flipped). The hindbrain and rostral spinal cord is outlined by the white dashed line. Arrowhead marks obex. **(B)** Comparing the dorsoventral soma positions of identified neuron groups [red: dINs, dINrs; blue: ascending interneurons (aINs), cINs; green: MNs, number of neurons given next to names] to the CHX10 immuno-positive cells (brown). The bottom of the hindbrain/spinal cord is set as 0 μm in the dorsoventral dimension and the mid/hindbrain border is set as 0 μm in the longitudinal dimension. *Indicates significance at *p* < 0.05 and ** at *p* < 0.01.

In zebrafish, excitatory interneurons in the swimming circuit express the transcription factor CHX10. Is it possible that the dINs in the tadpole swimming network are also CHX10 positive? We previously used CHX10 antibody to stain the neurons in the tadpole CNS (Figure 6A, Roberts et al., 2012). The vast majority of the CHX10 positive cells are located in the ventral quarter of the hindbrain and ventral half of the spinal cord. Although there were many CHX10 positive cells in the rostral hindbrain region, the staining was relatively light. The density of CHX10 positive cells decreased at the caudal end of the hindbrain and further in the spinal cord. We compared the distributions of these CHX10 positive cells to those for neurons identified physiologically and anatomically within similar longitudinal regions. The somata of sensory interneurons in the mechanosensory pathways are very dorsal so we excluded them in this type of comparison (Roberts et al., 2010). CHX10 positive cells are more ventral than cINs (*n* = 11, *p* < 0.01) and aINs (*n* = 7, *p* < 0.05) but have similar dorsoventral locations to that for dINs (*n* = 33), MNs (*n* = 15) and the repetitive-firing descending interneurons (dINrs, *n* = 13, all independent median test, Figure 6B), which are intensively active during tadpole struggling activity (Li et al., 2007). The CHX10 positive cells are unlikely to include the MNs since MNs have not been shown to be CHX10 positive in any vertebrate (Goulding, 2009; Gosgnach, 2011). However, these comparisons suggest that both dINs and dINrs may form part of the CHX10 distribution.

## Discussion

Interrogating the roles of individual groups of neurons in the vertebrate locomotor CPG has been difficult, even in model animals with largely identified neural circuits (Brownstone and Wilson, 2008). From a simple perspective, locomotion can be initiated by activating a single or a small number of “command” cells by sensory information, descending commands from higher brain regions or experimentally. Among invertebrates for example, the activation of serotonin command cells in the marine Mollusc *Clione limacina* (Arshavsky et al., 1992), trigger cells in sea slug *Tritonia diomedea* (Frost et al., 2001), in leech (Brodfuehrer and Friesen, 1986) or in crayfish (Edwards et al., 1999) can all initiate swimming. These “command” cells receive and process sensory information and pass the swimming decision to the CPG to start locomotion. They then either remain silent or fire tonically during swimming.

In the relatively simple tadpole CNS we have recorded from several powerful dINs, among a large number of random recordings, whose activation showed behavior like that of invertebrate “command” cells by repeatedly leading to swimming. Almost invariably, this only occurred after removal of mid- and forebrain. The reason is unclear, but it may be that the transections in the hindbrain cut off a subpopulation of dINs located rostral to the transection, causing some homeostatic upregulation of dIN synaptic strengths or neuronal excitability (Cooke and Parker, 2009) that increased the likelihood of individual dINs showing powerful effects in the caudal end. Our analyses suggest that the ability of a single dIN to initiate swimming requires recruitment of a wider dIN population. We previously reported extensive electrical coupling among hindbrain dINs. However, the electrical coupling alone has never been strong enough to evoke postsynaptic dIN firing in paired recordings (Li et al., 2009). The initiation of swimming thus is very likely due to dIN chemical synapse connections (Li et al., 2004b). In this way, the initial dIN excitation can be amplified by the positive feedback connections among dINs (Li et al., 2006) allowing it to start swimming network activity. More detailed analyses of the recordings provided some indirect support for our explanation and the timing of what happens between the initial spiking of powerful dINs and the initiation of swimming: the recruitment of a population of dINs to drive the swimming CPG. However, unlike the silence or un-patterned activity of command cells during locomotion in invertebrates (Brodfuehrer and Friesen, 1986; Arshavsky et al., 1992; Frost et al., 2001), dINs fire very reliably on each swimming cycle (Figure 1B). Therefore, dINs are not like the invertebrate locomotor “command” cells in simply providing an initial command. Instead, dINs are the core CPG neurons in the tadpole swimming circuit. Swimming is initiated when the dIN population is recruited to fire and maintained dIN firing is essential for sustaining the swimming rhythm (Moult et al., 2013).

How the decision for swimming is made after sensory excitation, however, is not completely clear at this stage, although the dIN is a clear candidate (Koutsikou et al., 2018). Once the mechanosensory neurons innervating trunk or head skin are excited, they directly excite sensory interneurons in the dorsolateral part of the spinal cord or in the trigeminal nuclei (Roberts et al., 2010; Buhl et al., 2012, 2015). The broad distribution of EPSP delays that summate to threshold and give rise to the first synchronous dIN firing cannot be explained by the very brief and short-latency firing of sensory pathway neurons. This suggests the existence of some intermediate interneurons upstream to dINs, producing small EPSPs, which require significant summation to reach dIN firing threshold. Therefore, they should be physiologically different from the usually powerful “command” cells in invertebrates discussed above. In swimming started by powerful dINs, swimming frequency should be lower due to the absence of sensory excitation at the beginning of episodes (Figure 3, Li and Moult, 2012). In mammalian spinal circuit, locomotion central pattern generation is proposed to consist of two layers: rhythm generation and pattern-formation (e.g., see Brownstone and Wilson, 2008). There is evidence that some tadpole brainstem neurons have un-patterned firing following skin stimulation, which extends sensory pathway activity and underlie the initial excitation of dINs before swimming starts. However, these interneurons do not appear to be required in the maintenance of swimming rhythms. This is because they do not receive excitation from the rhythmically active dINs (Koutsikou et al., 2018) and we show in this article that dINs can initiate swimming without the activation of sensory pathways. Therefore, a rhythm generating layer is unlikely present between the sensory initiation pathway and the tadpole swimming CPG. Instead, both swimming rhythm-generation, which is based on rebound firing in dINs after reciprocal inhibition (Li et al., 2006; Soffe et al., 2009), and the basic left-right alternation of activity are mediated by the same identified CPG circuit (Figure 1B).

Like other types of CPG neurons, dINs fires in a one-spike-per-cycle manner during swimming. How do we determine that dINs drive the swimming rhythms rather than them driven by other neurons? We previously compared the timing of phasic EPSCs dINs received during swimming and their spiking time and found that in the caudal hindbrain and rostral spinal cord region, dIN spiking often preceded the onset of EPSCs. This suggests that dIN firing is not driven directly by the fast EPSPs they receive, but likely from the rebound following reciprocal inhibition (Soffe et al., 2009). In line with dINs driving the swimming CPG, dINs spike reliably earlier than other neurons at similar longitudinal positions. dINs’ role as the driving force for swimming was further confirmed by silencing experiments where large hyperpolarizing currents were injected into individual dINs in the caudal hindbrain region (Moult et al., 2013). These currents likely spread into neighboring dINs through electrical coupling, inhibited more dINs and stopped swimming. Physiology and anatomical data also support that dINs form a continuous column extending into the caudal hindbrain, where their ascending axon branches provide feedback excitation critical for the maintenance of swimming (Li et al., 2006). Interestingly, some V2a neurons recorded in the middle hindbrain of zebrafish are rhythmically active during fictive swimming (Kimura et al., 2013). The caudal hindbrain of adult lamprey has also been recently demonstrated with swimming rhythm generating capacity (Buchanan, 2018). It is well known that locomotion rhythm generation lies in the spinal cord of vertebrates. It remains an interesting question if this wider distribution of swimming CPG in both spinal cord and hindbrain is just a feature of a developing motor circuit, or it is common for swimming vertebrates.

Do dINs potentially possess molecular characteristics that could be used to link them to similar excitatory interneurons in the locomotion CPGs in other vertebrates? Apart from using immunocytochemical methods to reveal neurons with different neurotransmitters, several molecular markers have been used in the past to identify the sensory Rohon-Beard (RB) cells, MNs, the ascending inhibitory interneurons in *Xenopus* tadpoles (Borodinsky et al., 2004; Li et al., 2004a). Unfortunately, proper double-labeling was generally lacking to confirm the specificity for these markers in segregating neuronal groups defined anatomically or functionally, although the anatomical identification of RB cells is reliable. In this study, we have compared the dorsoventral location of CHX10 nuclei to the soma location for all known classes of ventrally located neurons. This has led to the conclusion that CHX10 is likely to be expressed in dINs and dINrs. Although MNs are located in similar, ventral locations, they have never been shown to express CHX10 in any vertebrate (Goulding, 2009; Gosgnach, 2011). Some dINrs are also located quite dorsally, implying the presence of some subgroups. Zebrafish CHX-10 expressing excitatory interneurons can be functionally divided into subgroups of bursting and regular-firing neurons based on their responses to intracellular current injections. Anatomically, the bursting subgroup only possesses descending axons (Song et al., 2018). Tadpole dINrs also only have descending axons but they fire repetitively. This may reflect differences among vertebrate species.

Unlike some invertebrate CPGs where inhibitory synapses and electrical synapses are common (Shepherd and Grillner, 2010), excitatory interneurons in the vertebrate locomotor CPG play critical roles. Such roles include providing the phasic and tonic excitation that drives the activity of the whole CPG, co-ordinating left-right motor activity, maintaining the locomotor rhythms (Kiehn, 2016) and potentially the propagation of rhythmic activity along the longitudinal body axis. In zebrafish, the CHX10-expressing interneurons also extend from the spinal cord into the hindbrain, although they appear to be more dorsally located in the hindbrain than the CHX10 positive cells in tadpoles (Kimura et al., 2006, 2013). Optogenetic tools have been developed in the fish to show that exciting V2a CHX10 neurons could start swimming and inhibiting them stopped swimming (Kimura et al., 2013; Ljunggren et al., 2014). A subpopulation of these CHX10 neurons in the ventromedial part of the hindbrain reticular formation appear be active only in the initial burst of swimming, contributing to the turning behavior in larval zebrafish (Huang et al., 2013). In mice, CHX10 have been shown to mark multiple functional groups of excitatory neurons in both the spinal cord and brainstem. Function of spinal CHX10 neurons in mouse seemed to be related to left-right motor activity alternation (Lundfald et al., 2007; Crone et al., 2008, 2009) and forelimb reaching (Pivetta et al., 2014). Surprisingly, optogenetically stimulating V2a neurons in the brainstem reticular formation in mice does not initiate locomotion, as suggested by an earlier study (Bretzner and Brownstone, 2013), but instead stops ongoing locomotion (Bouvier et al., 2015). Similar optogenetic tools to specifically target dINs as a neuronal group have not been developed. However, among a large number of recordings, we have fortunately found several examples of individual dINs, whose excitation could evoke swimming in this study. We previously showed that in many cases injecting large hyperpolarizing currents into individual dINs in the caudal hindbrain regions could slow down (Li and Moult, 2012) or stop ongoing swimming (Moult et al., 2013). Therefore, we conclude that dIN activity is both sufficient and necessary in tadpole swimming rhythms.

In conclusion, we have provided further evidence that hindbrain excitatory neurons, dINs, are vital in the initiation and maintenance of tadpole swimming. A key feature of the excitatory dIN population is that activity in some members or even, as here, single members, can recruit wider firing in the population. They also may express the transcription factor CHX10 in early development. There appears to be strong functional and anatomical similarity between CHX10 positive V2a neurons in zebrafish and dINs in tadpoles, i.e., swimming rhythm initiation and maintenance, but in mammals CHX10 neuronal functions in motor control have diversified.

## Data Availability

The datasets generated for this study are available on request to the corresponding author.

## Author Contributions

W-CL collected and analyzed data. W-CL and SS contributed to writing.

## Conflict of Interest Statement

The authors declare that the research was conducted in the absence of any commercial or financial relationships that could be construed as a potential conflict of interest.
